# ﻿*Columnea rubromarginata* (Gesneriaceae), a new species from the western Andean slopes of Colombia and Ecuador

**DOI:** 10.3897/phytokeys.268.174855

**Published:** 2025-12-29

**Authors:** John L. Clark, Sofía Ballesteros, Laura Clavijo

**Affiliations:** 1 Marie Selby Botanical Gardens, 1534 Mound St., Sarasota, FL 34236, USA Marie Selby Botanical Gardens Sarasota United States of America; 2 Universidad Nacional de Colombia–Sede Bogotá, Facultad de Ciencias, Instituto de Ciencias Naturales, Bogotá, D.C., 111321, Colombia Universidad Nacional de Colombia Bogotá Colombia

**Keywords:** Chocó Biogeographic Region, *
Columnea
*, Esmeraldas, Gesneriaceae, Reserva Chococito, taxonomy

## Abstract

Field expeditions to the western Andean slopes of Colombia and ongoing herbarium research resulted in the discovery of a new species of *Columnea* (Gesneriaceae). *Columnea rubromarginata* J.L.Clark & Clavijo, **sp. nov.** is described from the Chocó Biogeographic Region in the premontane wet forests of Esmeraldas in northern Ecuador and the departments of Chocó and Valle del Cauca in Colombia. The new species is vegetatively remarkable for the presence of a rarely observed broad dark red submarginal band on the abaxial leaf surface. Based on International Union for Conservation of Nature (IUCN) guidelines, a preliminary conservation status of Vulnerable (VU) is recommended for *C. rubromarginata*.

## Abstract (es)

Expediciones en las estribaciones occidentales de los Andes en Colombia y la curación de especímenes en herbarios resultaron en el descubrimiento de una nueva especie de *Columnea* (Gesneriaceae). *Columnea rubromarginata* J.L.Clark & Clavijo, **sp. nov.**, se describe de la Región Biogeográfica del Chocó en los bosques húmedos premontanos de Esmeraldas, en el norte de Ecuador, y de los departamentos de Chocó y Valle del Cauca en Colombia. La nueva especie es notable vegetativamente por la presencia de una rara y amplia banda submarginal de color rojo oscuro en la superficie abaxial de la hoja. Con base en las directrices de la UICN, se recomienda un estatus preliminar de conservación de Vulnerable (VU) para *C. rubromarginata*.

## ﻿Introduction

The family Gesneriaceae belongs to the order Lamiales and currently comprises more than 3,900 species distributed among 150 genera (Weber 2004; Weber et al. 2013, 2020; GRC 2025). Phylogenetic hypotheses based on molecular sequence data strongly support the classification of the family into three subfamilies and seven tribes (Weber et al. 2013, 2020), each representing well-supported monophyletic lineages (Ogutcen et al. 2021). In the Neotropics, most species occur in the subfamily Gesnerioideae, which includes over 1,200 species and 77 genera (Clark et al. 2020; GRC 2025). Within Gesnerioideae, *Columnea* L. belongs to the tribe Gesnerieae and subtribe Columneinae (Weber et al. 2013, 2020). A key feature that distinguishes *Columnea* from closely related genera is the presence of indehiscent berries, in contrast to the fleshy, bivalved capsules typical of closely related genera.

With more than 220 recognized species (Clark et al. 2020; GRC 2025), *Columnea* is the largest genus among the Neotropical members of the subfamily Gesnerioideae (Weber et al. 2013, 2020). Species of *Columnea* range from Mexico southward to Bolivia, with their greatest diversity occurring in the northern Andes of Colombia and Ecuador. In Colombia, the genus is exceptionally diverse, with at least 80 species recorded in the 1990s (Kvist et al. 1998), 95 species by 2016 (Clavijo et al. 2016), and a current richness of 106 species, including the taxon described here. *Columnea* was monographed for Ecuador by Kvist and Skog (1993), who recognized 57 accepted species. Since that treatment, 18 additional species have been described, and the inclusion of the species presented here increases the total diversity of *Columnea* in Ecuador to 75 species.

Molecular phylogenetic studies strongly support its monophyly (Clark et al. 2006; Smith et al. 2013; Schulte et al. 2014). However, most traditional subgeneric classifications are artificial and are not supported by molecular phylogenetic studies (Smith and Carroll 1997; Smith 2000; Clark and Zimmer 2003; Clark et al. 2006, 2012; Smith et al. 2013; Schulte et al. 2014). For this reason, the new species presented here is not classified or assigned to a subgeneric rank.

## ﻿Materials and methods

Plants were photographed in the field and subsequently pressed and dried. The type specimens are deposited in Colombia at the Herbario Nacional Colombiano (COL), Herbario de la Universidad del Valle (CUVC), Herbario de la Universidad de Antioquia (HUA), and Herbario del Jardín Botánico de Medellín (JAUM); in the United States at the Missouri Botanical Garden (MO), Marie Selby Botanical Gardens (SEL), New York Botanical Garden (NY), and the United States National Herbarium (US); and in Europe at the Conservatoire et Jardin Botaniques de la Ville de Genève (G). Photographs of live specimens were taken in the field using a Nikon D7500 DSLR with a Nikon 105 mm lens. Morphological observations and measurements were made from live collections, alcohol-preserved material, and digital images using the ImageJ program (https://imagej.nih.gov/ij/).

The extinction risk was assessed following the IUCN Red List Categories and Criteria (2012) and the updated criteria of the IUCN Standards and Petitions Committee (2024). Field observations and collection sites from fieldwork were used to evaluate the IUCN category. The extent of occurrence (EOO) and area of occupancy (AOO) were calculated using the software program GeoCAT (Bachman et al. 2011) with the default setting of 2 km, which corresponds to a 4 km^2^ grid cell.

## ﻿Taxonomic treatment

### ﻿ Columnea rubromarginata

Taxon classificationPlantaeLamialesGesneriaceae

J.L.Clark & Clavijo sp. nov.

C299E871-ADDB-5F58-AF24-41ACA9E54275

urn:lsid:ipni.org:names:77374378-1

Fig. 1

#### Type.

**Colombia** • Chocó, municipio San José del Palmar, western slopes of the Cordillera Occidental, Reserva Chococito, 4°52'45.99"N, 76°15'38.18"W, 800–950 m alt., 12 Aug. 2024 (fl. & imm. fr.), *John L. Clark, Esteban Barco, Cristhian Cardona, Camila Davila, Jeison Pineda, Catalina Delgado, Sofía Ballesteros, Nelson Salinas & Laura Clavijo 19345* (holotype: COL; isotypes: CUVC, G, HUA, JAUM, MO, NY, SEL! [barcode: SEL095528], US).

#### Diagnosis.

Vegetatively similar to *Columnea purpurimarginata* L.P.Kvist & L.E.Skog by the conspicuous, broad submarginal dark red band on the undersurface of the blades, but *C. rubromarginata* differs in having a red corolla tube with a yellow limb (vs. yellow corolla tube with white limb and red striations in *C. purpurimarginata*) and linear calyx lobes nearly equaling the corolla tube (vs. broadly ovate, short calyx lobes covering only the base of the corolla tube).

#### Description.

Epiphytic or climbing herb to subshrub. **Stem** scandent, branched, terete in cross-section, 3.0–6.5 mm diam., herbaceous to subwoody, green to yellow green, sparsely pilose at the base and densely pilose toward the apex, trichomes white, 2.0–3.0 mm long; internodes 1.5–3.2 cm long. **Leaves** opposite and anisophyllous; petiole of the larger leaf 0.3–0.7 cm long, terete, strigose, with multicellular, white trichomes, 2.0–3.0 mm long, becoming slightly hirsute when dry; larger blade oblanceolate, 13.5–27.0 × 3.5–7.6 cm, coriaceous, green adaxially, green abaxially with a dark red marginal band 2.0–7.3 mm wide, apex acuminate, dark red abaxially, base oblique and rounded, margin sparsely serrate, sparsely to densely pilose on upper and lower surfaces with multicellular, branched, whitish trichomes, margin and the apex with dark red trichomes, blades with 8–13 pairs of secondary veins; smaller blade sessile linear to lanceolate, 1.7–2.0 × 0.8–0.9 cm, 2–3 pairs of main lateral veins, often caducous. **Inflorescence** reduced to 1–3 axillary single axillary flowers; peduncles absent; bracts 7.5–10.0 × 1.0–1.7 mm, green with red apex, lanceolate, apex acuminate, base truncate, margin entire, both surfaces with multicellular, branched, whitish trichomes; pedicel erect 18–40 mm long, green, pilose, with multicellular, branched, transparent trichomes. **Calyx** lobes 5, fused basally for 1.9–2.5 mm, equal in length, mostly red on outer surface and green on inner surface, sometimes with the base green on outer and inner surfaces, 27.0–31.4 × 2.0–2.9 mm, persistent in fruit, secondary venation suppressed, linear, apex attenuate, base truncate, margin entire for basal half, with 4–8 teeth per side along the apical half, pilose on upper and lower surfaces with multicellular, branched, whitish trichomes. **Corolla** tubular, 34.0–39.0 mm long; tube erect relative to calyx, 6.3–6.5 mm wide at the middle, dark red and densely pilose with white branched trichomes, nectary chamber 4.0–7.0 × 3.0–5.0 mm, tube narrowed above the nectary chamber, throat 4.3–5.2 mm diam., limb yellow on inside and red with yellow margins on outside, corolla with 5 equal-sized lobes, 2.4–4.5 × 2.0–4.0 mm, straight, elliptic, apex rounded, margin entire, densely pilose abaxially, with white multicellular trichomes. **Androecium** of 4 didynamous stamens, filaments 23.0–32.0 mm long, adnate to the corolla tube for 3.5–5.5 mm, glabrous, staminode absent; anthers oblong, coherent, dehiscing by longitudinal slits, 2.2–2.5 × 2.4–2.6 mm. **Gynoecium** with a single-dorsal lobed nectary gland, ca. 2 mm long, glabrous; ovary superior, ca. 4.9 × 2.6 mm, ovate, sparsely pilose; style included, ca. 27 mm long, glabrous; stigma bilobed. **Fruit** a globose berry, 7.7–15.0 × 6.5–10.0 mm, ovoid, green, pilose with translucid trichomes; seeds elongate, fusiform, and brown, < 1 mm long.

#### Phenology.

Collected in flower in February, March, April, May, June, August, and October, and with fruits in August.

#### Etymology.

**Figure 1.**
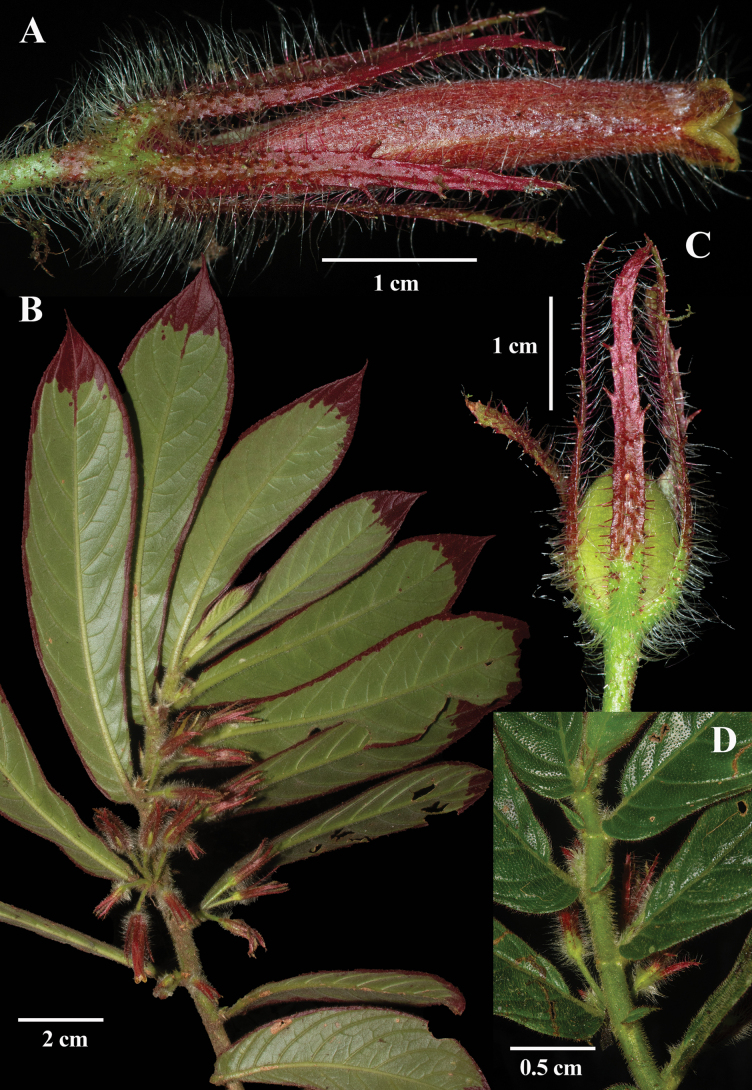
*Columnea rubromarginata* J.L.Clark & Clavijo. **A.** Flower; **B.** Habit; **C.** Immature fruit; **D.** Adaxial surface of shoots showing anisophyllous leaves (**A–D** from *J.L. Clark et al. 19345*). Photos by J.L. Clark.

The epithet *rubromarginata* is derived from the Latin *ruber* (red) and *marginatus* (bordered or edged), referring to the distinctive dark red submarginal band on the abaxial (underside) surface of the leaves (Fig. 1B).

#### Distribution and preliminary assessment of conservation status.

The geographic distribution of *Columnea rubromarginata* corresponds to the Chocó Biogeographic Region, an area characterized by relatively high levels of precipitation and epiphytic diversity, between 30 and 950 m in elevation (Gentry 1986; Pérez-Escobar et al. 2019). *Columnea rubromarginata* is locally abundant in the privately owned and operated Reserva Chococito in the Colombian Chocó Department along the western slopes of the Cordillera Occidental. It has also been documented along roads in the following Colombian localities along the western Andean slopes of the Cordillera Occidental: Chocó (highway Bolívar–Quibdó and Quibdó-Tutunendo) and Valle del Cauca (old highway Buenaventura–Cali). One population was also documented in the environs of Lita in northern Ecuador (Fig. 3).

The extent of occurrence (EOO) is calculated as 30,769.253 km^2^, corresponding to a Near Threatened status; the area of occupancy (AOO) is calculated as 28 km^2^, which is within the threshold for Endangered status under subcriterion B2. The only known collection from Ecuador dates to 1987, prior to construction of the highway between Lita and Esmeraldas, and it is likely that this population has been extirpated. Fewer than ten collections are known, meeting subcriterion B2a for Vulnerable. If the Ecuadorian record is excluded from the GeoCAT analysis, the recalculated EOO is 11,805.300 km^2^, corresponding to a status of Vulnerable under criterion B1, and an AOO of 24 km^2^, which falls within the threshold for Endangered under criterion B2.

The species is threatened in Ecuador and Colombia by ongoing habitat loss and fragmentation resulting from deforestation, the expansion of agriculture (especially African palm plantations), mining and illegal logging (especially in Ecuador), and urbanization. These threats were observed by the authors between 1994 and 2022 and have led to a recent decline in the quality and extent of suitable habitat.

The preliminary IUCN category is therefore suggested as Vulnerable (VU), based on subcriteria B1, B2a,b(i,ii,iii).

#### Comments.

**Figure 2.**
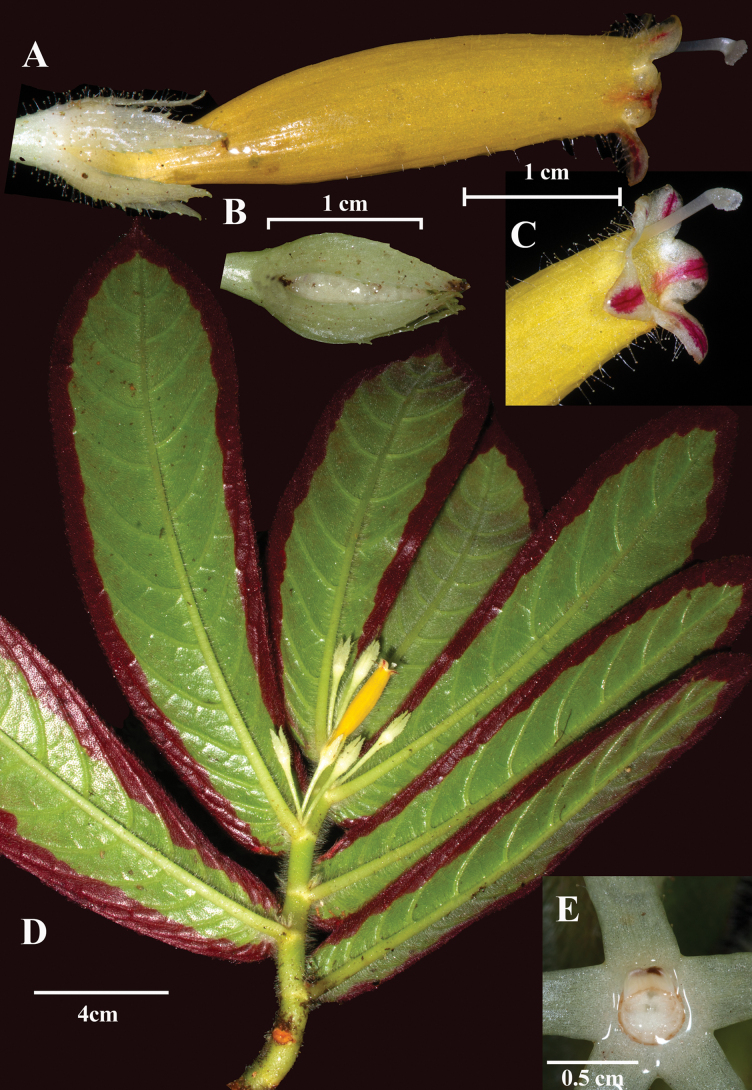
*Columnea purpurimarginata* L.P.Kvist & L.E.Skog. **A.** Flower; **B.** Immature fruit; **C.** Corolla lobes; **D.** Habit; **E.** Nectary gland (**A, B** from *J.L. Clark et al. 17224*; **C–E** from *J.L. Clark et al. 11086*). Photos by J.L. Clark.

Most epiphytic species of *Columnea* with dorsiventral shoots have abaxial leaf surfaces with red apices or entirely red leaves. In one species, *C. eubracteata* Mansf., the leaves are mostly red with green apices. *Columnea rubromarginata* is remarkable for its broad red band along the abaxial surface of the leaf margins (Fig. 1B). The only other species with a similar red marginal band is *C. purpurimarginata* L.P. Kvist & L.E. Skog (Fig. 2; see also fig. 8 in Kvist and Skog 1993), a species for which several specimens are now recognized as *C. rubromarginata*. Vegetatively, these two species appear nearly identical, but they are readily distinguished by their corollas and calyx lobes. In *C. rubromarginata*, the calyx lobes are linear, uniformly dark red, and more than 2.7 cm long, or nearly as long as the corolla tube (Fig. 1A, C). In contrast, in *C. purpurimarginata*, the calyx lobes are uniformly pale green, ovate, and less than 1 cm long, covering only the basal third of the corolla tube (Fig. 2A, B). Both species have elongated tubular corollas but differ in their color patterns. *Columnea rubromarginata* has a uniformly red corolla with a yellow limb (Fig. 1A), whereas *C. purpurimarginata* has a uniformly yellow corolla with a white limb and red striations on the corolla lobes (Fig. 2A, C).

**Figure 3.**
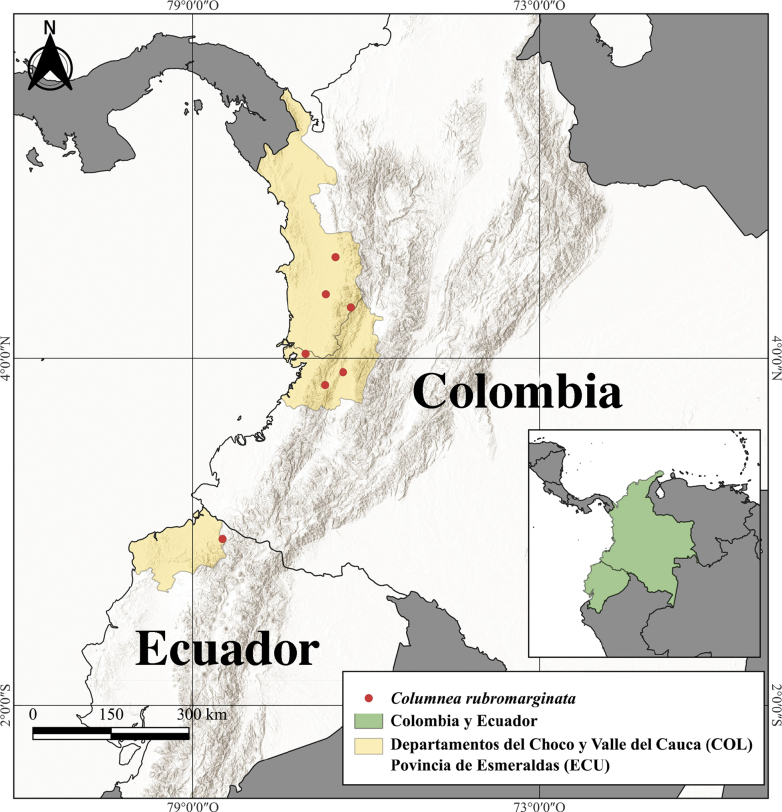
Distribution map of *Columnea rubromarginata* indicating documented localities in Colombia and Ecuador.

*Columnea rubromarginata* and *C. purpurimarginata* share overlapping geographic and ecological habitats. Both species are more abundant in Colombia, but the type locality for *C. purpurimarginata* is in Ecuador, and it has been documented several times in northern Esmeraldas during expeditions conducted between 2000 and 2022. In contrast, the type locality of *C. rubromarginata* is in Colombia, and this species has been documented only once in Ecuador, in 1987 (*H. Wiehler 87104*).

Another vegetatively similar species is *Columnea pulcherrima* C.V. Morton, but in this species the marginal red band is much narrower and often absent, giving the leaves an almost uniformly green appearance or green with red apices. Additionally, the leaves of *C. pulcherrima* are thinner and more membranaceous compared to the thicker, more coriaceous leaf blades of *C. rubromarginata*.

#### Specimens examined.

**Colombia. Choco**: • road Bolivar−Quibdo, 18 km NE of Tutunendo, near Vereda 21, at Alto de Veinte, hacienda of Ruben Jaramillo, half hour walk downhill from road and farm house, 580 m, 15 Mar 1987 (fl.), *H. Wiehler & GRF Expedition of 1987 8723* (SEL, US); • Andagoya, 70–100 m, 20–30 Apr 1939 (fl.), *E.P. Killip 35051* (BM, US); • Negría, dense forest along Río San Juan, 50 m, 17 Apr 1939 (fl.), *E.P. Killip 35024* (BM, US); • between La Oveja and Quibdó, 1 Apr 1931 (fl.), *W.A. Archer 1765* (US); • Quibdó, entre Quibdó y Tutunendo, carretera a Carmen de Atrato 8.1 km de Quibdó, 110 m, 8 Aug 1982 (fr), *L. Albert de Escobar et al. 2160* (CUVC, HUA). **Valle del Cauca**: • old road from Cali to Buenaventura, about 10 km beyond Queremal, on path down to Río Anchicayá, 30 Apr 1972 (fl.), *H. Wiehler et al. 7289* (SEL, US); • road Cali-Buenaventura, near Río Zabaletas, 2 May 1972 (fl.), *H. Wiehler et al. 72140* (SEL, US); • Sabaletas, km 29 of highway from Buenaventura to Cali, 25 m, 4 June 1944 (fl.), *E.P. Killip & J. Cuatrecasas 38751* (F, US); • Río Cajambre, San Isidro, 5–100 m, 2–5 May 1944 (fl.), *J. Cuatrecasas 17354* (F, SEL, US); • Río Calima, La Trojita, 5–50 m, Feb–Mar 1944 (fl.), *J. Cuatrecasas 16821* (F, US); • Buenaventura, Corregimiento Bajo Calima, Vereda San Isidro. km 39 of road to Bahia Malaga (under construction), zona de explotación forestal (Cartón de Colombia), Frente A35 (Hans), 4.033333°N, 76.96667°W, 30–50 m, 18 May 1989, *D.C. Daly 6041* (CUVC); • Buenaventura, margen derecha aguas abajo Río Anchicaya; por trocha que desde el pueblo San José de Anchicaya, conduce al pueblo Calle Larga, 19 m, 19 Oct 2012 (fl), *A. Giraldo 7941* (CUVC). **Ecuador. Esmeraldas**: • forest near Lita, 550−650 m, 6 May 1987 (fl.), *H. Wiehler 87104* (SEL).

## Supplementary Material

XML Treatment for Columnea rubromarginata

## ﻿Additional information

### Use of AI

No use of AI was reported.

### Author ORCIDs

John L. Clark https://orcid.org/0000-0002-1414-6380

Sofía Ballesteros https://orcid.org/0009-0005-1007-7735

Laura Clavijo https://orcid.org/0000-0002-3009-9158

### Data availability

All of the data that support the findings of this study are available in the main text.
